# Generalised power graph compression reveals dominant relationship patterns in complex networks

**DOI:** 10.1038/srep04385

**Published:** 2014-03-25

**Authors:** Sebastian E. Ahnert

**Affiliations:** 1 Theory of Condensed Matter, Cavendish Laboratory, University of Cambridge, JJ Thomson Avenue, Cambridge CB3 0HE, United Kingdom

## Abstract

We introduce a framework for the discovery of dominant relationship patterns in complex networks, by compressing the networks into power graphs with overlapping power nodes. When paired with enrichment analysis of node classification terms, the most compressible sets of edges provide a highly informative sketch of the dominant relationship patterns that define the network. In addition, this procedure also gives rise to a novel, link-based definition of overlapping node communities in which nodes are defined by their relationships with sets of other nodes, rather than through connections within the community. We show that this completely general approach can be applied to undirected, directed, and bipartite networks, yielding valuable insights into the large-scale structure of real-world networks, including social networks and food webs. Our approach therefore provides a novel way in which network architecture can be studied, defined and classified.

Since the field of complex networks research emerged a little more than a decade ago^1^,^2^,^3^, a plethora of network measures has been proposed to capture different aspects of network complexity^4^. Many of these aim to identify “communities” of nodes^5^, which are typically defined as sets of nodes that are more densely interconnected than they are connected to nodes outside the set^6^. These include blockmodels^7^, modular decomposition^8^, dynamical approaches based on random walks^9^ or synchronisation^10^, as well as information-theoretic methods^11^,^12^. The computational cost and optimisation of these methods has also received considerable attention^13^. While many definitions of communities only consider disjoint sets of nodes, some approaches, particularly in the more recent literature, allow overlapping communities. Such methods include clique percolation^14^, spin models^15^, stochastic mixed-membership block models^16^, latent attribute models^17^, and methods based on spectral clustering^18^. power graphs^19^ and link communities^20^,^21^ also allow overlaps by focusing on sets of links, rather than nodes, that can be grouped together. In many cases the overlap arises from ‘fuzzy' or stochastic membership of nodes in communities^15^,^16^,^17^,^18^. But even among the approaches that avoid a notion of uncertainty to define overlapping communities, existing approaches impose restrictions on the nature of the overlap, for instance by fixing the topology of the connectivity between overlapping nodes^14^, prohibiting power nodes that overlap but are not complete subsets^19^, or only grouping links connected to a particular node^21^. Our aim is to transcend these definitions by proposing a completely general way of identifying dominant relationship structures in networks through lossless compression of networks into power graphs. Importantly, this approach places no constraints on the overlap between sets of nodes. This link-based approach encompasses traditional notions of network communities, such as the partition of a network into densely interconnected subsets of nodes, but at the same time offers a much more general definition of ‘community', as a set of nodes that is connected to another set of nodes in the same way. The removal of constraints on the overlap between communities gives rise to a vast space of possible node sets. The problem of selecting among these is solved by performing a global compression of the network.

A power graph is a representation of a conventional graph in which the power nodes are sets of conventional nodes, and in which the poweredges between them signify that all nodes in one power node are connected all nodes in another power node. In the existing literature power nodes have been non-overlapping or subsets of each other^19^. Here we introduce a framework without such constraints, allowing power nodes to be non-overlapping or to overlap in the most general way (see Figure 1). In order to describe a poweredge between power nodes A and B we need to specify the nodes in each of the power nodes. If there are *N* nodes in total, *n_A_* nodes in power node A, and *n_B_* nodes in power node B, then the information required to describe the poweredge (assuming at most *N* power nodes) is: 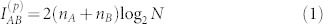
By contrast, the information required to specify all *n_A_n_B_* edges that connect the *n_A_* nodes in A with the *n_B_* nodes in B is: 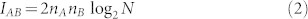
By rewriting the edges between the nodes in A and B as a single poweredge we can compress the amount of information require to describe this set of edges by 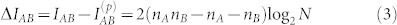
Note that these expressions, like all that follow below in the main part of the paper, are valid for directed and bipartite networks. The expression for undirected networks is given in the Methods section. We can now successively compress sets of edges by defining pairs of node sets such as A and B above. In the following we will refer to such a pair of node sets as a *compressible component* of the graph, if Δ*I_AB_* > 0. We do not impose any constraints on the membership of nodes in these sets, so that nodes can be in one or both sets of one or multiple compressible components. A given edge can therefore also appear in more than one compressible component. When compressing a graph into multiple compressible components we need to take this into account if we want to calculate the overall compression achieved. For details of calculating the overlap, and of the greedy algorithm used for the overall compression through successive selection of compressible components, see the Methods section.

In order to assign meaning to the overlapping power nodes we use term enrichment analysis. If a set of properties is associated with every node, we can compare the distribution of same properties of the nodes in a given set with the distribution of the properties expected by chance. We can thus characterise a power node using the node properties that occur significantly more often than expected by chance. This procedure is used for sets of genes in the context of Gene Ontology^22^, where it is known as GO Term Enrichment Analysis. For more details, see the Methods section.

## Results

### Compressibility of real-world networks

**Figure 2:**
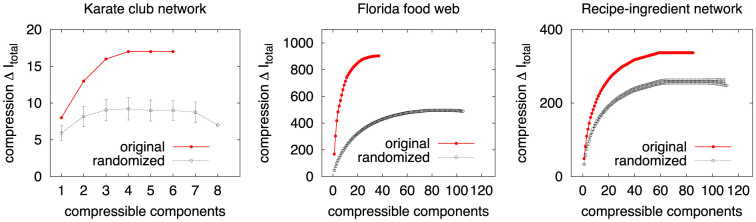
Compressibility of three real-world networks (the undirected Karate club social network^23^, the directed Florida food web network^24^, and a bipartite recipe-ingredient network derived from an online recipe database^25^), in each case compared to the compressibility of 100 randomized networks with the same degree distribution. In all three cases the real-world networks are significantly more compressible than their random counterparts.

We apply our compression to three very different real-world networks: An undirected social network, a directed food web and a bipartite networks of recipes and the ingredients they contain. The first question that might arise in this context is whether these real-world networks are more compressible than one might expect by chance. Figure 2 shows the overall compressibility Δ*I_total_* (see Methods) for a given number of compressible components in both the real-world networks and, for each these, 100 randomized networks with the same degree distributions. From this it is clear that the real-world networks are far more compressible than their randomized counterparts. Below we describe these three networks and their compressible components in much more detail.

### Social networks

**Figure 3:**
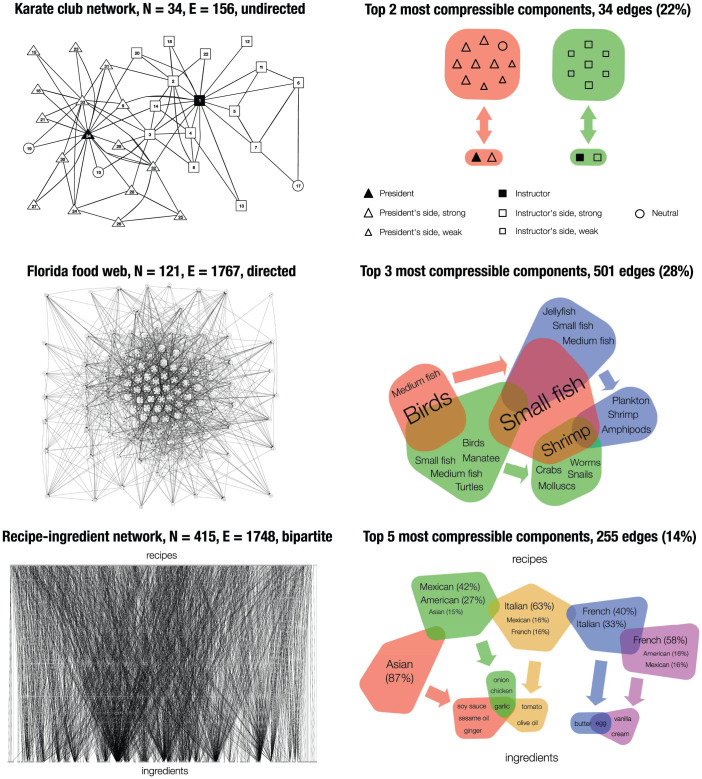
Finding the compressible components of three real-world networks. TOP: The Karate club network consists of two distinct communities. Unlike conventional community detection algorithms, the compressible components not only describe the two separate factions present in the network, but also identify the nodes that constitute the leaders of the two communities. MIDDLE: The Florida food web provides a particularly good example for the uses of compressible components, as the conventional definition of communities as sets of nodes that are densely connected with each other does not yield useful sets of nodes in food webs. Compressible components identify sets of predators that are unified by the type of prey they seek out. Note also that the three compressible components correspond to three different habitats: Organisms interacting across the air/water interface (red), organisms living in water (blue) and organisms living in or on the water/ground interface (green). BOTTOM: The most compressible components of a bipartite network of ingredients and recipes reveal the usage patterns of ingredient combinations in different cuisines, even though the information on cuisines is completely unknown to the compression algorithm. The partial overlap between the power nodes shows the relative proximity of cuisines to each other.

The social network of a karate club studied by Zachary^23^ (34 nodes, 78 edges) has become a well-known benchmark data set for community detection. The reason is that the social network split during the course of the study due to an internal dispute between members of the club. This provides a clear partition of nodes, which can be compared to the predictions of community detection algorithms that are run on the original network. Our aim is to go beyond the classical problem of partitioning the graph into communities. As explained above, compressible components are much more than a community detection algorithm. This is demonstrated by the first two compressible components of the karate club network (Fig. 3a), which not only show a very clear partition of nodes (as the two components are entirely non-overlapping) but also identify the leaders of the two factions (nodes 1, the Instructor, and 34, the President) very clearly, who, in each case together with one other node, are connected to many of the club members on their side of the dispute.

Term enrichment analysis confirms that the alignment of the two large power nodes with their leaders is statistically significant, as the Bonferroni-corrected p-values are 1.43E-02 (President's faction) and 2.79E-03 (Instructor's faction).

### Food webs

Food webs are networks of predator-prey relationships between biological species. Here we study the food web of a Florida ecosystem^24^ encompassing 1767 interactions between 122 types of organism. The framework of compressible components is particularly apt for the context of a food web, as similar organisms in a food web are unlikely to form predator-prey relationships with each other, and therefore unlikely to be identified as communities by an approach that compares the edge density within a community with inter-community edge densities. Organisms in a food web can be defined by the combinations of predator-prey relationships that they take part in, as can be seen in Fig. 3b, which shows the three most compressible components in the Florida food web. Interestingly, these components also represent interactions between and within the three primary environments in this ecosystem: air, water, and the ground, or bottom of the sea. The first component shows us that a large variety of birds all feed on a large variety of small fish. The second shows us that some birds and fish, as well as turtles and manatee feed on small animals in or on the seabed, such as worms, molluscs, snails and crabs. The third shows us that an almost entirely disjoint set of fish from this feeds on amphipods, plankton and shrimp, which drift or swim in the water.

Term enrichment analysis confirms the significance of these dominant link relationships: Small fish (Bonferroni-corrected p-value: 8.30E-06) are eaten by large birds (1.59E-03) and medium birds (5.49E-04); Worms (8.08E-03) and Shrimp (3.1E-02) are eaten by medium birds (8.83E-03); and Copepods (4.14E-04) and Crustaceans (1.81E-02) are eaten by small fish (1.50E-07).

### Recipe-ingredient networks

Recipes for food preparation and the ingredients they require form a bipartite network that has received attention in the recent network literature^25^,^26^. One of the attractions of this data set is the availability of external classifiers in the form of cuisines. From the online recipe database Epicurious (http://www.epicurious.com) we randomly selected 50 recipes from the five largest cuisines, giving us 249 recipes (as one selected recipe had two cuisine identifiers, and was selected twice), which contain 116 ingredients. The resulting bipartite network consists of 1748 edges. The five most compressible components, representing 255 edges, or 14% of the network, are shown in Fig. 3c and are each dominated by recipes from the cuisine of a particular country or region. The ingredient power nodes identify key ingredients of these cuisines.

Term enrichment analysis confirms the cuisine-specificity of the power nodes. The Bonferroni-corrected p-values for the enrichment of the dominant cuisines are (going from left to right through the power nodes in Fig. 3c): 9.97E-06 (Asian), 1.90E-02 (Mexican), 1.05E-03 (Italian), 6.17E-02 (French), and 2.82E-02 (French).

## Discussion

The results outlined above demonstrate the way in which compressible components differ from other, superficially similar approaches. Conventional community detection approaches would have for instance found the two communities in the karate network, but would have failed to extract the pairs of nodes associated with the leadership of these communities. A method for detecting bicliques might have picked up the result found in the ingredient-recipe network, but such methods are tailored to bipartite networks, whereas compressible components can be applied to any network, whether it happens to be bipartite or not. But it is the food web example that is particularly pertinent, as the traditional notion of community structures as densely connected subgraphs in the network fails when it comes to food webs. Meaningful sets of predator and prey species are defined by connections that are *external* to these sets, and compressible components offer a single, general framework that can highlight such relationships between sets of nodes, as well as identify a diverse array of more traditional node communities.

This method is robust against perturbations of the network. If we remove an arbitrary edge that forms part of a compressible component with node sets *A* and *B*, then the Δ*I_AB_* of that compressible component will be changed by 2 log_2_*N* (min(*n_A_*, *n_B_*) − 1), which is approximately equal to 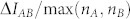, which is particularly small if *n_A_* and *n_B_* are significantly different, which is often the case. The rank ordering of compressible components is therefore unlikely to be changed by any given random perturbation of the network topology.

At present the computational cost of this method is high, scaling approximately as 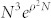 for a network of *N* nodes and link density *ρ*, defined as the fraction of total node pairs that are connected by edges. For sparse networks that fulfill *ρ*^2^*N* < 1 (or equivalently 〈*k*〉^2^/*N* < 1, where 〈*k*〉 is the average degree) networks of up to a few thousand nodes are feasible on a desktop machine, with the computational cost scaling roughly as *N*^3^.

In conclusion we have introduced a framework for the compression of networks into power graphs with overlapping power nodes. This method can be applied to undirected, directed and bipartite networks, and offers a way to identify dominant relationships in the network as well as a completely general way of defining overlapping node communities. Possible extensions of this approach could be to include exceptions to the requirement that all nodes in one set are connected to all nodes in the other. A more advanced generalisation could be to apply this methodology to weighted networks.

## Methods

### Compressible components

The amount of information required to specify the nodes in the power nodes A and B is: 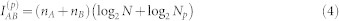
where *N_p_* is the number of power nodes. If we have at most *N* power nodes we can bound this by: 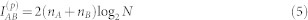
By contrast, the information required to specify all *n_A_n_B_* edges that connect the *n_A_* nodes in A with the *n_B_* nodes in B, in a directed or bipartite network, is: 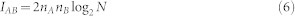
In an undirected network this is: 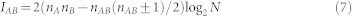
where the ± distinguishes the cases in which self-interactions are allowed (−) or not (+), and where *n_AB_* is the number of nodes (if there are any) that are in both power nodes. The *n_AB_*(*n_AB_* ± 1)/2 term is necessary to avoid double-counting undirected connections between any two nodes that appear in both sets *n_A_* and *n_B_*. If there are *n_AB_* such nodes then we need to subtract *n_AB_*(*n_AB_* ± 1)/2 from the total 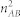 connections that are regarded as part of the *n_A_n_B_* term. So by rewriting the edges between the nodes in A and B as a single poweredge we can compress the amount of information require to describe this set of edges by 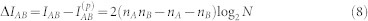
for directed or bipartite networks, and 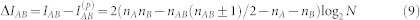
for undirected networks with (−) or without (+) self-interactions permitted. We can now successively compress sets of edges by defining pairs of node sets such as A and B above. In the following we will refer to such a pair of node sets as a *compressible component* of the graph, if Δ*I_AB_* > 0. We do not impose any constraints on the membership of nodes in these sets, so that nodes can be in one or both sets of one or multiple compressible components. A given edge can also appear in more than one compressible component. When compressing a graph into multiple compressible components we need to take this into account if we want to calculate the overall compression achieved. If there are *M* possible compressible components for an adjacency matrix *M* we can write each compressible component in terms of binary membership vectors 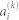, 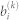 over the nodes *i*, where *k* = 1..*M*. If we now consider a set *S* of compressible components, the adjacency matrix *m_ij_* can be decomposed into outer products of the compressible component vectors, the remaining edges in the network and an overlap correction: 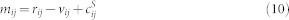
where 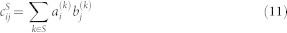
It follows that the overlap *v_ij_* can be defined by 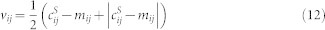
in which the entries denote the number of times an edge is repeated in the compressible components (in other words, the number of times it appears, minus one). The remainder *r_ij_*, representing the uncompressed edges, can be defined by 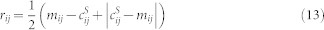
For undirected networks with self-interactions the term 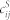 in the above formulae for *m_ij_*, *r_ij_*, and *v_ij_* becomes 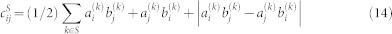
and for undirected networks without self-interactions: 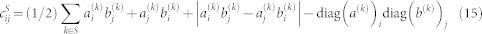
Writing **M**, **V**, and **R** for these quantities, **a** and **b** for the membership vectors, and denoting an *N*-dimensional vector of 1s as **1**, we can therefore write for a given compressible component *k* with membership vectors **a**^(*k*)^ and **b**^(*k*)^: 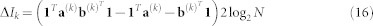
for directed or bipartite networks, and as: 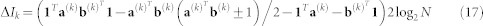
for undirected networks with (−) or without (+) self-interactions. With the overlap **V** defined for a given set *S* of compressible components as above, we can write the total compression as: 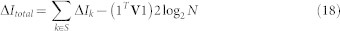
for directed or bipartite networks, and as: 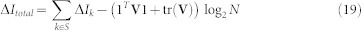
for undirected networks (with or without self-interactions). Our aim will be to maximise Δ*I_total_* over all possible sets of compressible components. We do this by first calculating all possible Δ*I_k_* and then employ a greedy algorithm to combine them, giving a Δ*I_total_*. The calculation of all possible Δ*I_k_* is achieved by iterating over all possible pairs of nodes and storing those pairs that share two or more neighbors in common (which gives Δ*I_k_* ≥ 0). The next iteration combines each of these pairs with an additional single node further on in the node iteration sequence. Triplets which share two or more neighbors are stored. The next iteration combines each triplet with an additional node, and stores these quadruplets if they are compressible. We therefore obtain a list of compressible components with compression values Δ*I_k_*. The greedy algorithm used to combine these chooses the compressible component with the largest Δ*I_k_* and then calculates the Δ*I_total_* for this component combined with each of the other components, choosing the second component that maximises the Δ*I_total_* for the two. The next step is to calculate the Δ*I_total_* for these first two components and each of the remaining components, again choosing that component which maximises Δ*I_total_* for all three. This continues until further addition of compressible components does not increase Δ*I_total_*.

### Enrichment analysis

If a set of properties is associated with every node, we can compare the distribution of properties of nodes in a given set with the distribution of the properties expected by chance. We can thus characterise a set of nodes using the node properties that occur significantly more often than expected by chance. This procedure is used in the context of Gene Ontology, where it is known as Term Enrichment Analysis^22^. A given property *i* which occurs *n_i_* times in *N* nodes appears *k* times in a set of size *s* with probability 
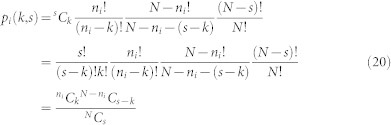
From this a p-value of statistical significance can be calculated by considering all values of *k* that are as likely or less likely to happen than a given k*: 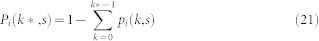
If we are considering a total of *T* properties, we are in effect testing *T* multiple hypotheses. To account for this, we apply the Bonferroni correction to the p-value P_i_(k*, *s*) to give us a Bonferroni-corrected p-value of: 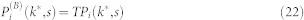
Note that this correction assumes independence of the T hypotheses. In cases where this assumption is likely to be flawed we can also calculate a corrected p-value by considering a degree- and partition-preserving randomisation of the bipartite membership network of nodes and sets.

## Author Contributions

S.E.A. designed the research, performed the analysis, and wrote the paper.

## Figures and Tables

**Figure 1:**
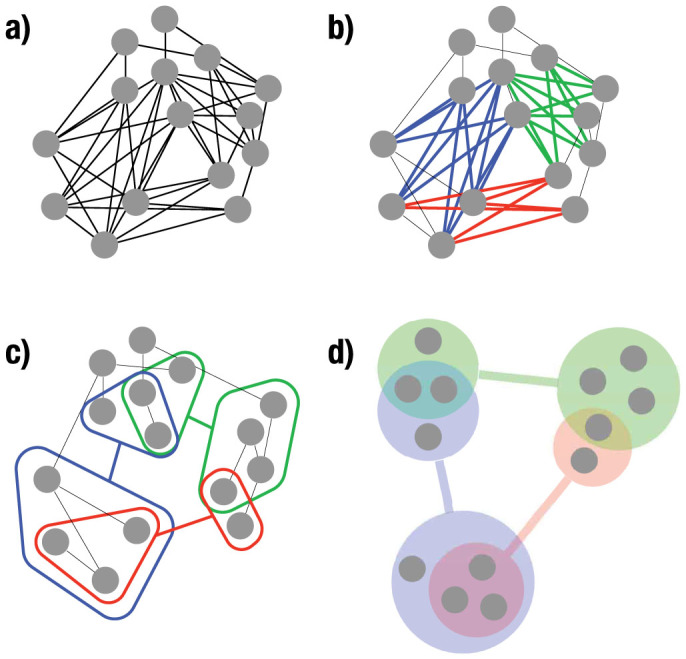
Illustration of the compression of a network into a power graph with overlapping power nodes. A poweredges between two power nodes signifies that all nodes in one power node are connected to all nodes in the other power node. The poweredges selected by our greedy algorithm, which successively compresses the network, are called compressible components. The original network is shown in (a), the edges that will be compressed are highlighted in (b), and the corresponding poweredges in (c). The final panel shows only these three most compressible components, as a simplified representation of the dominant relationship structures in the network. The three poweredges represent 30 edges in the original network, or 71% of the total edges.
